# An Overview of the 2009 A(H1N1) Pandemic in Europe: Efficiency of the Vaccination and Healthcare Strategies

**DOI:** 10.1155/2016/5965836

**Published:** 2016-03-13

**Authors:** Funda Samanlioglu, Ayse Humeyra Bilge

**Affiliations:** Faculty of Engineering and Natural Sciences, Kadir Has University, 34083 Istanbul, Turkey

## Abstract

2009 A(H1N1) data for 13 European countries obtained from the weekly influenza surveillance overview (WISO) reports of European Centre for Disease Prevention and Control (ECDC) in the form of weekly cumulative fatalities are analyzed. The variability of relative fatalities is explained by the health index of analyzed countries. Vaccination and healthcare practices as reported in the literature are used to explain the departures from this model. The timing of the vaccination with respect to the peak of the epidemic and its role in the efficiency of the vaccination is discussed. Simulations are used to show that on-time vaccination reduces considerably the final value of *R*(*t*), *R*_*f*_, but it has little effect on the shape of normalized curve *R*(*t*)/*R*_*f*_.

## 1. Introduction

The 2009 A(H1N1) pandemic was a major influenza pandemic that caused global alert. It was a variant of 1918 influenza that caused millions of fatalities. All countries applied some type of intervention and vaccines were developed but it turned out that the pandemic was not as deadly as anticipated and vaccination campaigns were not as effective as planned in most of the countries. As summarized in Section 2.1, a large number of research papers addressed various aspects of the pandemic: basic parameters were measured from clinical information and review articles on the healthcare measures and on epidemiological research were published for various countries.

In this paper, we study the 2009 A(H1N1) pandemic in 13 European countries, based on weekly influenza surveillance overview (WISO) reports published by European Centre for Disease Prevention and Control (ECDC) [1]. The official pandemic period for A(H1N1) is from week 18 of 2009 to week 35 of 2010 and the formal end is declared as week 32 of 2010 [2]. Here we study the fatality data for the so-called second wave (or autumn/winter wave), from week 36 of 2009 to week 15 of 2010. In the following, for practical purposes, we will count weeks from the beginning of 2009, hence our data will cover the period from week 36 to week 68.

The aim of the present work is to study the inference of the epidemic parameters from fatality data only, as discussed in our previous work [3]. We show that the scatter in relative fatalities can be explained by the healthcare measures and we use pulse vaccination simulations for the Susceptible-Infected-Removed (SIR) model to measure the effects of timing of vaccinations.

## 2. Preliminaries

### 2.1. Literature Survey

In the literature there are a number of papers devoted to the study of 2009 A(H1N1) pandemic in a single country such as Turkey [4], Denmark [5], Canada [6], Iran [7], Morocco [8], and Mexico [9] or to a comparative study [10–14]. Several others focus on the transmission dynamics of the pandemic, providing estimates of “basic reproduction number,” “incubation period (latent period),” “generation time,” and “serial interval” as below.

The “basic reproduction number” (*R*_0_) is the average number of secondary cases generated from a single infected case in a population with no immunity to the disease and in the absence of interventions to control the infection. The “incubation period” is defined as the time between infection and symptom onset while the “latent period” is defined as the time of being infected and becoming infectious. The latent period is the notion that is relevant in epidemiological dynamics but for influenza type diseases the latent period and incubation period are used synonymously. The “generation time (interval)” is the average delay between the time of infection of a case and the time of infection of secondary cases infected by that case; and the “serial interval” is defined as the difference between the onset of symptoms of the primary and secondary cases [15, 16]. The serial interval is more easily observable than the generation time; however generation time is more relevant in the epidemic spread. For influenza type diseases, the distinction is not crucial. For A(H1N1), the mean incubation period is estimated as 1.4 days (95% confidence interval (CI), 1.0–1.8); the mean generation time of the pandemic is estimated as 2.5 to 3 days, and the serial interval is estimated as 2.2 to 2.3 days [11, 17]. Since *R*_0_ depends on the contact rate which may differ from country to country, the estimate of *R*_0_ has certain spread. For example, it is estimated as 1.1–1.4 in United Kingdom [12], 1.8 (95% CI, 1.5–2.2) in United States [17], 1.3–1.4 in Brazil [12], 1.4–1.6 in Mexico [18], 1.2–1.6 in Peru [19], 1.8–2.1 in Thailand [20], 1.2–1.5 in Australia [12], and 1.2–1.4 in Chili [12]. A review of studies presenting estimates of transmission parameters of the 2009 A(H1N1) pandemic is given in Boëlle et al.'s [13] work, where they show that the mean generation time of 2009 A(H1N1) pandemic was lower than the median for 1889, 1918, 1957, and 1968 influenza pandemics; and the median reproduction number was similar to 1968 pandemic and slightly smaller than 1889, 1918, and 1957 pandemics.

### 2.2. Preprocessing of the Data

Data collected for the European Union and European Economic Area (EU/EEA) WISO includes sentinel syndromic surveillance of influenza-like illness (ILI) and acute respiratory infection (ARI) and virological surveillance data, hospital-based sentinel surveillance of severe acute respiratory infection (SARI) data, and qualitative reporting data as well as influenza deaths. Data related to weakly influenza deaths includes case based deaths resulting from severe acute respiratory infection (SARI) and weakly aggregated influenza deaths reported by countries, which is also complemented by active monitoring of official websites for deaths [2, 21]. The first WISO report, published on 15.09.2009, includes the data of week 36 of 2009. Our study covers the period from week 36 of 2009 to week 15 of 2010 (or from week 36 to week 68 counted cumulatively for practical purposes) called the “second wave.” In Table 1, we present 33 weeks of cumulative fatality data, from September 2009 to May 2010, of 13 different European countries, obtained from WISO reports.

Fatality data related to weeks 44, 45, and 52 were not available in WISO reports; linear interpolation was used to fill the missing values. It has been reported that the weekly mortality reports might be unreliable due to reporting delays [2].

**Figure 1:**
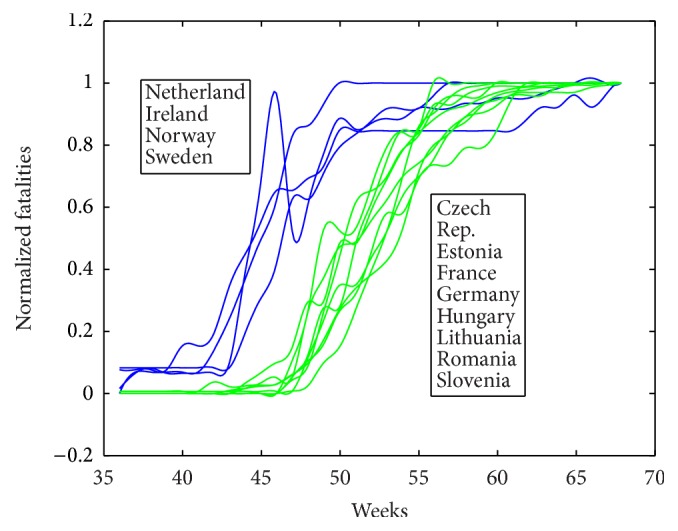
Normalized fatalities for analyzed European countries.

The time series for fatalities for the analyzed countries are presented in Figure 1. From this figure we can see that the epidemic starts earlier in Netherlands, Ireland, Norway, and Sweden and later in Czech Republic, Estonia, France, Germany, Hungary, Lithuania, Romania, and Slovenia. The reason of this early start-up may be the early start of the influenza season due to climate in Northern countries.

### 2.3. Demographic Structure and Healthcare Measures

Geographic and demographic information of various European countries is presented in Table 2 [22]. This piece of information is used to normalize and compare the number of fatalities in different countries. The age structure of the population is also a key issue since the 2009 A(H1N1) pandemic is characterized with low infection rate among people over the age of 60 presumably due to their prior exposure to antigenically related influenza viruses, resulting in the development of cross-protective antibodies [2, 23]. As opposed to seasonal influenza, during the 2009 A(H1N1) pandemic, 80% of fatalities were within the age group under 65, and about 25%–30% of fatalities were among healthy adults that were not considered as part of risk groups [2]. It is reported by several studies [14, 24, 25] that during the 2009 A(H1N1) pandemic, the proportion of fatalities among the young increased in comparison to seasonal influenza deaths. In fact, Van Kerkhove et al. [26] reported that globally the median age was 46 among fatalities. We have included information on age structure for the countries we analyzed in Table 2; however since their age structure was more or less homogeneous we overlooked this information and decided to use total figures. In this table, Human Development Index (HDI) and health index (HI) of countries are also presented along with average latitudes. The census data and average latitudes are obtained from CIA (*The World Factbook*) [22] and* Eurostat Yearbook* [27]; HDI and HI values are acquired from Human Development Reports [28]. HI published in the framework of the United Nations Development Program [28] is one of the objective measures of the efficiency of the healthcare system. HDI that includes HI as a component can also be considered as an alternative [28]. HDI is a measure of human development, and it has three basic dimensions: a long and healthy life (health index), access to knowledge (education index), and a decent standard of living (income index). The HDI value is calculated as the geometric mean of normalized indices measuring achievements in each dimension.

During the 2009 A(H1N1) pandemic, several pharmaceutical (antivirals, vaccination) and nonpharmaceutical (school closures, travel restrictions, limiting public gatherings, etc.) measures were recommended across communities [29, 30]. All countries agreed on EU Health Security Committee (HSC) recommendations to immunize risk and target groups such as healthcare workers, pregnant women, and those older than six months with chronic ill health; however some countries even targeted children or entire population [2, 10]. Hungary was the first EU country able to start vaccination (during week 40), and other countries followed afterwards. In EU/EEA, at least 46.2 million (9% of the population) was vaccinated as of mid-July 2010 [2].

Vaccination coverage of various European countries is presented in Table 3 based on Mereckiene et al.'s [10] study. The vaccination coverage data for Lithuania was not available and presented data related to Germany corresponds to the vaccinated people above the age of 14.

In this table, *t*_*i*_ and *t*_*e*_ denote, respectively, the onset and the end of the epidemic wave which are estimated as the week before the first fatality and the week after the last fatality, *t*_*e*_ being counted from the beginning of 2009. The values *t*_1_ and *t*_2_ denote the weeks at which vaccination starts and ends, as reported in [10], *t*_2_ being counted cumulatively. Latest reported time is week 86 corresponding to the end of the survey. The duration of the epidemic wave, Δ*T*, is defined as Δ*T* = *t*_*e*_ − *t*_*i*_, with *t*_*i*_ and *t*_*e*_ estimated in Table 3. The time span between the onset of the epidemic pulse and the starting of the pulse vaccination Δ*V* is defined as Δ*V* = *t*_1_ − *t*_*i*_. *QV* = Δ*V*/Δ*T* is the relative timing of the vaccination campaign within the epidemic pulse and a negative or small positive value indicates on-time vaccination campaign. *QV* together with the total vaccination percentage *V*_*f*_ will be considered as a measure of the efficiency of the vaccination strategy. In many countries, vaccination timing goes beyond the end of the epidemic but presumably the vaccination rate drops towards the end of the epidemic and the vaccination percentage saturates. Thus we will assume that vaccination is practically terminated at the end of the epidemic as if pulse vaccination was applied.

### 2.4. SIR and SEIR Epidemic Models with Vaccination

Compartmental models in epidemiology are based on the subdivision of the individuals in a society into distinct groups with respect to their status regarding the disease. The basic compartmental models are the Susceptible-Infected-Removed (SIR) and the Susceptible-Exposed-Infected-Removed (SEIR) models that represent quite adequately the spread of an epidemic in a society where the total population is constant, the characteristics of the disease are time independent, and no vaccination policy is in force. In these models, it is further assumed that immunity, once acquired, cannot be lost; hence the passage among the compartments is one-directional. This situation fits well with the spread of seasonal epidemics in a homogeneous closed society.

The standard Susceptible-Infected-Removed (SIR) and Susceptible-Exposed-Infected-Removed (SEIR) models [31, 32] consist of differential equations governing the dynamics of a population where the individuals can be “Susceptible” (*S*), “Exposed” (*E*), “Infected” (*I*), and “Removed” (*R*). Vaccination is incorporated in the model by adding the group of “Vaccinated” (*V*) individuals who gain immunity without going through an infectious period. We reserved the term “Removed” to the group of individuals who gain immunity after going through an infectious period.

The resulting differential equations for the SIR and the SEIR system with vaccination are given as

(1) dSdt=βSI−νSt,dIdt=βSI−ηI,dRdt=ηI,dVdt=νSt,dEdt=βSI−εE,dIdt=εE−ηI.

In these equations, the parameters *β*, *ε*, *η*, and *ν* are constants. In the SIR and SEIR models, the ratio of the parameters *β*/*η* turns out to be equal to the basic reproduction number *R*_0_, when a first-order approximation is used for *I*(*t*) [33, 34].

The reciprocals of the parameters *η* and *ε* are, respectively, the infection period and the incubation period (latent period), respectively. The parameter *ν* is the vaccination rate; hence models without vaccination are obtained by putting *ν* = 0. Since the total population is assumed to be constant, the normalization conditions are *S* + *I* + *R* + *V* = 1 and *S* + *E* + *I* + *R* + *V* = 1.

### 2.5. Exact Solutions for Pulse Vaccination

The differential equations for the SIR system with or without vaccination are solved implicitly for *I* and *S* as

(2) $$\begin{array}{c} \left(\;I-I_{i}\;\right)+\left(\;S-S_{i}\;\right)+\left(\;\frac{\nu }{\beta }\;\right)\ln\left(\;\frac{I}{I_{i}}\;\right)-\left(\;\frac{\eta }{\beta }\;\right)\ln\left(\;\frac{S}{S_{i}}\;\right)=0, \end{array}$$

where *S*_*i*_ and *I*_*i*_ ≠ 0 are the initial values of *S* and *I*, respectively. For the SEIR system without vaccination we have a similar relation:

(3) $$\begin{array}{c} \left(\;E+I\;\right)-\left(\;E_{i}+I_{i}\;\right)+\left(\;S-S_{i}\;\right)-\left(\;\frac{\eta }{\beta }\;\right)\ln\left(\;\frac{S}{S_{i}}\;\right)=0, \end{array}$$

where *E*_*i*_ is the initial value of *E*. The SEIR system with vaccination is an essentially third-order system that could not be integrated as in the case of the SIR system with vaccination.

In the following we assume that vaccination starts at *t* = *t*_1_ and stops at *t* = *t*_2_. The conditions as *t* → −*∞* are characterized by *S* → 1, *I* → 0, *E* → 0, and *R* → 0; hence the initial conditions should be specified according to *I*_*i*_ + *E*_*i*_ + *S*_*i*_ − (*η*/*β*)ln⁡(*S*_*i*_) = 1. It follows that at the initial stage prior to vaccination the implicit relations for the SIR and the SEIR models are, respectively,

(4) I+S−ηβln⁡S=1,E+I+S−ηβln⁡S=1,

regardless of the initial conditions. Let *S*_*f*_, *R*_*f*_, and *V*_*f*_ be the final proportions of Susceptible, Removed, and Vaccinated individuals, respectively. Since the final state is characterized by *I* = *E* = 0, for both models the implicit relations are reduced to

(5) $$\begin{array}{c} S_{f}-\left(\;\frac{\eta }{\beta }\;\right)\ln\left(\;S_{f}\;\right)=1. \end{array}$$

It follows that the basic reproduction number *R*_0_ = *β*/*η* is expressed in terms of *S*_*f*_ as

(6) $$\begin{array}{c} R_{0}=\frac{\beta }{\eta }=-\frac{\ln\left(1-R_{f}-V_{f}\right)}{\left(R_{f}+V_{f}\right)}=-\frac{\ln\left(S_{f}\right)}{1-S_{f}}, \end{array}$$

regardless of the vaccination coverage. If vaccination has never been applied, *S*_*f*_ = 1 − *R*_*f*_, while if pulse vaccination has been in effect, *S*_*f*_ = 1 − *R*_*f*_ − *V*_*f*_. Thus in the case of pulse vaccination, *R*_0_ can be obtained by knowing the total percentage of Removed and Vaccinated individuals.

## 3. The Effects of Healthcare Quality of Countries

The basic parameter of the epidemic *R*_0_ and the final proportion of the Removed individuals *R*_*f*_ in the SIR and SEIR models are related by a one-to-one nonlinear relationship. Thus the basic reproduction number that can be measured from clinical studies at the early phases of an epidemic can also be found from the total proportion of Removed individuals at the postepidemic phase. The difficulty here lies in the fact that the final proportion of Removed individuals is hard to estimate. Nevertheless, the total number of fatalities can be considered as a measure of the individuals affected by the disease. The proportion of individuals who die from a disease is known as the case fatality rate (CFR). In the case of an influenza-like illness, the case fatality rate possibly depends on the quality of healthcare. The purpose of this section is to study the effects of healthcare, specifically, the relation between the relative fatalities and the healthcare indices for the countries that we study.

**Figure 2:**
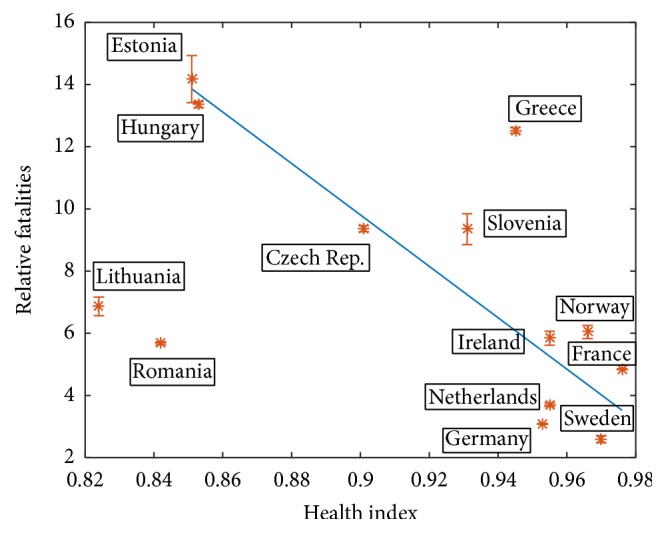
Relative fatalities versus the health index.

In order to examine the correlations between the relative fatalities and HDI and HI values, associated correlation coefficients are calculated. Weak negative correlations are found based on correlation coefficients of −0.4386 and −0.4834, respectively. Relative fatalities (*D*_*f*_/*N*)10^3^ versus the health index (HI) are shown in Figure 2, which displays roughly this negative correlation, despite numerous exceptions that will be discussed. In preliminary work, we have studied the effect of both indices and we have seen that for the countries under consideration they are closely correlated and we decided to work with HI values of the countries.

In this figure, the linear fit is obtained by minimizing the number of outliers with trial and error method. The countries that lie well off the linear fit are Lithuania and Romania with lower than expected relative fatalities and Greece with higher than expected relative fatalities. These countries are considered as outliers with the minimum error of 2.9%.

At the right lower part of the graph, corresponding to high HI, we observe that the relative fatalities are lower for Germany compared to France and lower for Sweden compared to Norway. Furthermore, the relative fatalities of Netherlands are also well below the regression line. In the next subsections, we discuss these relations.

### 3.1. Discussion of the Results for Netherlands

The time evolution of the data has excessive fluctuations but we may consider the total number of fatalities data reliable. From Table 3, we can see that vaccination timing was appropriate and the coverage was as high as 30%. This may explain the low relative fatalities but we should also take into account the fact that Netherlands is the most densely populated country among the ones analyzed and the dependency of the parameter *β* on the population density may have a saturation effect.

### 3.2. Comparison of the Results for Germany and France

Merler et al. [35] reported that the peak of the pandemic was delayed in France due to timing of the school holidays (weeks 44 and 45) and the peak was predicted to happen on average at week 43.6 but actually happened at week 49. We can see that although Germany and France have similar demographic structures and vaccination policies and even though France has higher HI, the relative fatalities of France were higher than Germany. Detailed vaccination policies and strategies followed by France are presented in Schwarzinger et al.'s [36] study. The difference can be explained by epidemic-specific precautions and healthcare procedures applied in Germany as reported in [37]. Wilking et al. [37] suggested that mortality in Germany due to 2009 A(H1N1) pandemic seems to have been one of the lowest fatality ratios in Europe and early treatment might have had an impact on overall mortality.

### 3.3. Comparison of the Results for Norway and Sweden

Norway and Sweden have similar geographic, demographic, and social characteristics. The difference between Sweden and Norway can be explained by their vaccination strategies. From Table 3, we can see that although vaccination started almost at the same time in both countries, for Norway it was almost 1/3 of the epidemic pulse, but for Sweden it was right at the beginning. It has actually been reported that in Norway vaccination campaign started too late to be effective [38] although probably above 40% of the Norwegian population got vaccinated [39]. In the study of de Blasio et al. [38], the effect of vaccination timing and sales of antivirals in Norway is analyzed with an age-structured SEIR model, and it is indicated that the countermeasures only prevented 11-12% of the potential cases relative to an unmitigated pandemic, and if the vaccination campaign would have started 6 weeks earlier, rather than week 43/2009, it is estimated that the vaccination alone might have reduced the clinical attack rate by 50%.

### 3.4. Vaccination Timing and Coverage of Analyzed Countries

**Figure 3:**
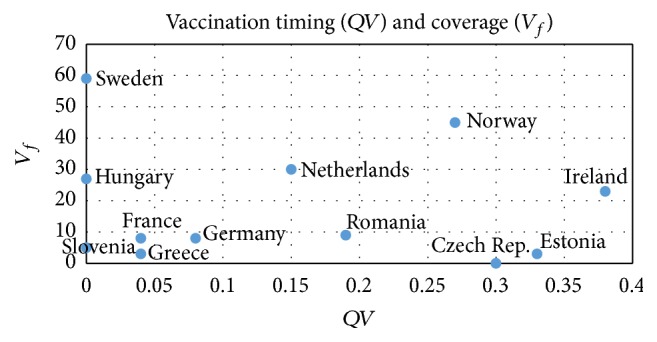
Vaccination timing versus vaccination coverage.

In Figure 3, vaccination timing (*QV*) versus vaccination coverage percentage (*V*_*f*_) are shown for each analyzed country.

In this figure, lower right corner corresponds to late vaccination campaigns with low percentage coverage. The ones at the upper right correspond to late vaccination and high coverage so these are relatively inefficient campaigns. The ones at the upper left are the most efficient with on-time vaccination campaigns and high coverage. This figure explains the difference between Sweden and Norway. Both countries have similar HI, and their geographic and demographic properties are similar, the absolute timing difference for starting vaccination is just 1 week but the relative difference is large, and this reflects to the burden of the epidemic.

**Figure 4:**
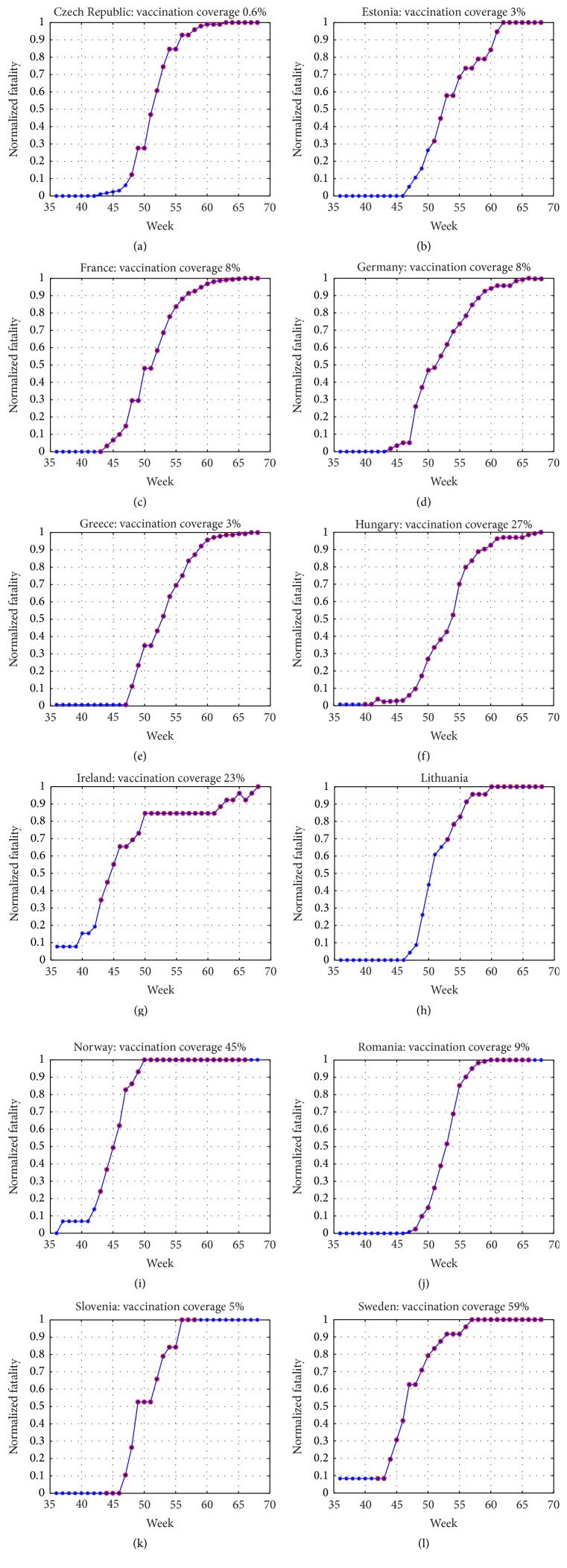
The normalized fatality data and the vaccination timings.

In Figures 4(a)–4(l), we present the data for each country and the vaccination timings, based on the vaccination information given in Table 3. Many countries claim having continued vaccination past the epidemic wave but the number of vaccinated people as a function of time is not given. It is reasonable to assume that the majority of the people have been vaccinated during the epidemic wave and vaccination continues only for specific target groups.

The timing of the vaccination should be measured by its location in the epidemic wave, as indicated in Table 3. For an efficient vaccination campaign, the ratio *QV* should be small, even negative. We see that in many countries the ratio *QV* is too high to be effective. From Table 3, we see that vaccination campaigns should have been most effective in Hungary, Sweden, and Netherlands. In Figures 4(a)–4(l), we can see this effect clearly for Sweden and Netherlands but not for Hungary.

## 4. Simulations for Pulse Vaccination Strategies

In this section, we present simulations for vaccination coverage and timing to conclude that on-time vaccinations have a considerable impact in reducing the final value *R*_*f*_, but vaccination effects are practically unobservable in normalized time evolution curves *R*(*t*)/*R*_*f*_.

In Table 3, the latest reported week is 86, corresponding to the end of the survey, but our study stops at week 68. The temporal distribution of vaccination rates is not given in these reports. However, it is reasonable that mass vaccination campaigns would be discontinued after the stabilization of the number of fatalities which signals the end of epidemic. In fact, the vaccination rates for France [10] confirm this. We thus assumed that total vaccination ratios are achieved by the end of week 68. Even if vaccination goes beyond the stabilization of *R*(*t*), it does not change *R*_*f*_; it simply decreases *S*_*f*_ to zero.

### 4.1. The Effect of Very Low Vaccination Coverage

**Figure 5:**
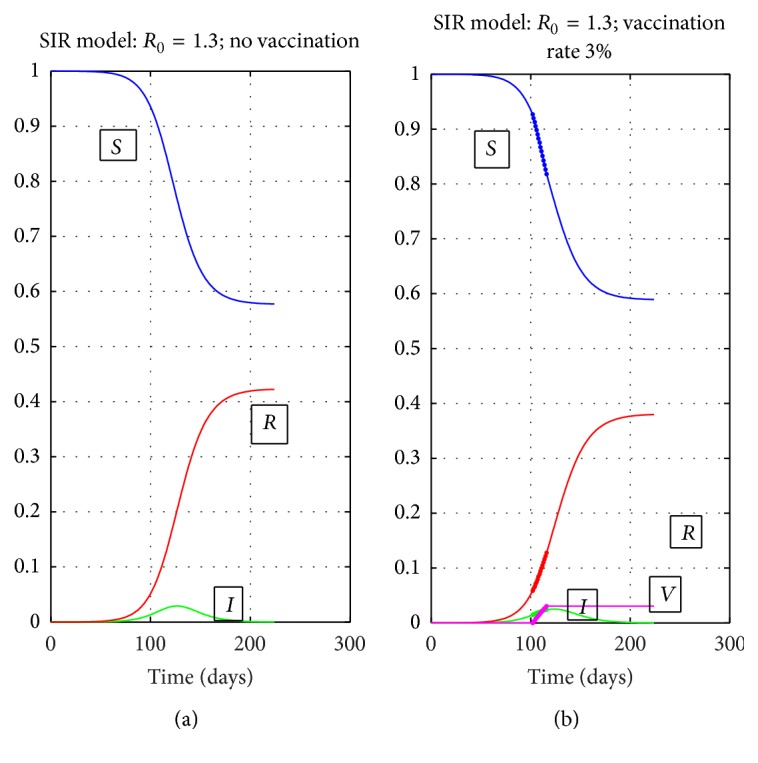
Comparison of no vaccination (a) and 3% vaccination (b) for the SIR model.

The total vaccination coverage given in Table 3 shows that total percentage of Vaccinated individuals was as low as 3% except for Hungary, Ireland, Netherlands, Norway, and Sweden. A comparison of the no vaccination and 3% vaccination for the SIR model is shown in Figures 5(a)-5(b).

In this simulation, vaccination starts when *I*(*t*) reaches half of its peak value and it is applied for 14 days. The final value of *S*(*t*) is more or less the same, but the final value of *R*(*t*) is lower. This issue is discussed in some detail in [40], where it is shown that the predicted number of cases of infections decreases linearly with vaccination coverage. Based on this, we considered vaccination to be effective on *R*_*f*_ only for Hungary, Ireland, Netherlands, Norway, and Sweden, where the coverage was above 20%.

### 4.2. The Effect of Vaccination Timing

It is well known that the timing of pulse vaccination is crucial in controlling the spread of the infection. It is reported that the progression of the epidemic is from west to east, as seen from Figure 1 where we present the timing of the epidemic. We also note that it started earlier in Norway compared to Sweden and this had a crucial effect on the efficiency of vaccination [2]. In Table 3, the onset of the epidemic wave is considered as the week before the first fatality and the end of the epidemic as the week after the stabilization of *R*(*t*). We thus measure “early” or “late” vaccination by the location of the starting time of the vaccination within this epidemic wave period.

**Figure 6:**
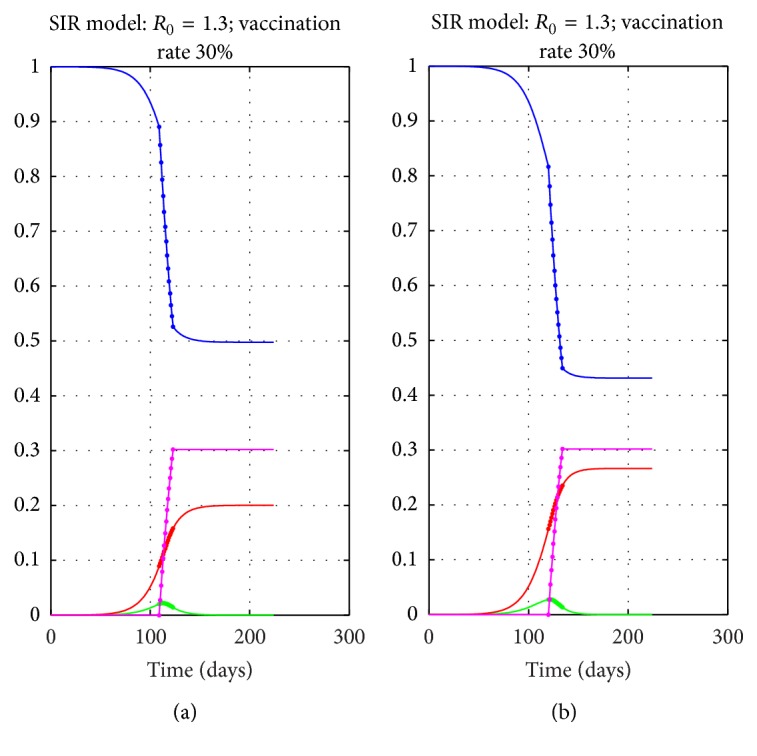
Comparison of early (a) and late (b) timings for 30% vaccination for the SIR model.

In Figures 6(a)-6(b) we present a simulation of 30% vaccination, starting “early” and “late.” The terms early and late refer to the timing of the vaccination with respect to the time *t*_*m*_ where *I*(*t*) for the no vaccination model reaches its maximum value. In our simulations, we used early and late pulse vaccinations as the ones starting one week earlier or later than *t*_*m*_. The reductions in *R*_*f*_ for each case show the importance of the vaccination timing.

Here we see that vaccination that starts late has little effect in reducing the number of Removed individuals. Vaccination that continues beyond the stabilization of *R*(*t*) is useless for influenza type epidemics. The simulations also show that even 2-week or 4-week campaigns may be sufficient.

### 4.3. The Effect of Vaccination on Normalized Curves

**Figure 7:**
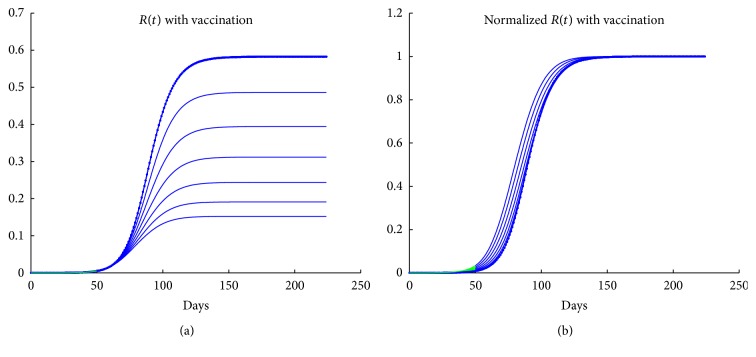
The effect of vaccination on actual (a) and normalized (b) *R*(*t*).

Although the efficiency of the vaccination on reducing the burden of the epidemic is unquestionable, it was a surprise to see that it had little effect on the shape of the time evolution curve, *R*(*t*). In Figures 7(a)-7(b), we present the actual and normalized time evolution curves *R*(*t*) and *R*(*t*)/*R*_*f*_ for various vaccination coverage percentages, ranging from no vaccination (top and right) to 50% vaccination. From Figure 7(b), we see that the effect of high vaccination coverage on the normalized curves is a back-shift in time, rather than a distinguishable change in the shape. From these figures, we see that vaccination at low rates is practically unobservable in normalized curves. Even at high rates, it appears as a shift and a reduction in the curvature of the first turn if it is applied early and a reduction of the curvature of the second turn, if it is applied late.

### 4.4. The Efficiency of Vaccination Campaigns

In order to compare the efficiency of various vaccination campaigns, we ran a pulse vaccination simulation using SIR model. The simulation runs over 3 parameters, the duration of the vaccination campaign, the onset of the campaign, and the percentage of Vaccinated individuals. For each of these cases, we ran the SIR model with pulse vaccination using representative parameters *R*_0_ = 1.5, *η* = 1/4 and we computed the final percentage of Removed individuals *R*_*f*_ as a function of these 3 parameters.

**Figure 8:**
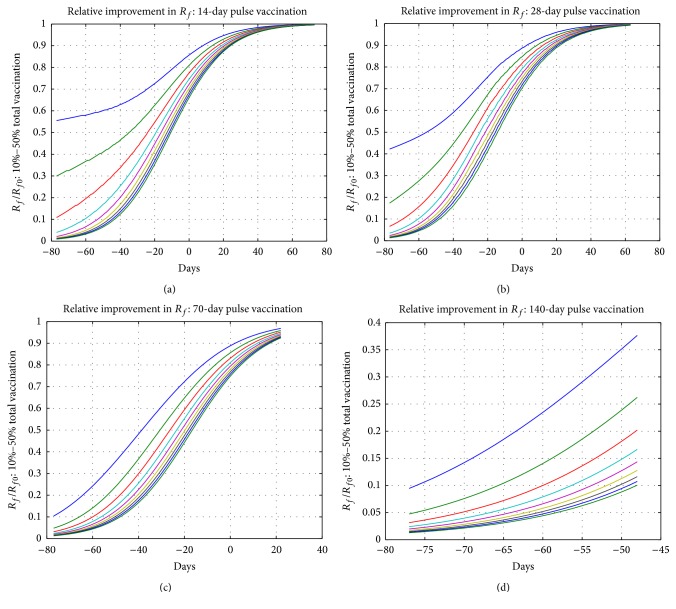
The improvement in *R*_*f*_ for total vaccination ratios ranging from 10% (top curves) to 50% in steps of 5% for the SIR model. Time origin is chosen as the peak of *I*(*t*) with no vaccination. Pulse vaccination starts at day *j* (horizontal axis) and lasts for 14 days (a), 28 days (b), 70 days (c), and 140 days (d).

We have chosen the duration of the pulse vaccinations as *k* = 14, 28, 70, and 140 days, as presented, respectively, in Figures 8(a)–8(d). In these figures, the curves from top to down correspond to vaccination ratios ranging from 10% to 50% in steps of 5%, respectively. Points of these curves are the ratio of the final percentage of Removed individuals with pulse vaccination (*R*_*f*_) and without pulse vaccination (*R*_*f*0_). The horizontal axis is day *j* of the onset of the vaccination campaign and the time origin is chosen at the peak *I*(*t*) without vaccination. As an example, the top curve in Figure 8(a) corresponds to a 14-day campaign with 10% vaccination ratio and one can see that a campaign that starts about 40 days before the expected peak of the epidemic reduces the final percentage of individuals affected by the epidemic to approximately 60% of this value when no vaccination is applied.

These figures can be useful in decisions related to vaccination strategies. For example, a short (*k* = 14) but early (*j* = −80) campaign with low coverage (15%) is as efficient as a long (*k* = 70) but relatively late (*j* = −40) campaign with higher (20%) coverage, both leading to approximately 30% improvement. On the other hand, campaigns with duration *k* = 70 that start later than day *j* = −30 can never reach this improvement level. Thus, vaccination campaigns should start as early as possible* with respect to the expected peak of the epidemic* and one should be aware that longer campaigns that start late would have limited efficiency despite their higher coverages.

## 5. Discussion

We have studied the relation between the HI and the relative fatalities of countries and obtained a linear fit by minimizing the outliers with trial and error method. We realized a roughly negative correlation and Lithuania, Romania, and Greece were considered outliers. Netherlands had lower relative fatalities than expected and this may be due to appropriate timing, high coverage of vaccination, and the saturation effect of the parameter *β* on the high population density of Netherlands. The relative fatalities in France were higher than in Germany although they have similar demographic structures and vaccination policies and the difference may be explained by epidemic-specific precautions and healthcare procedures applied by Germany. Norway had higher relative fatalities than Sweden although they are demographically and HI-wise similar, and this can be explained by vaccination strategies, specifically by the timing of the vaccination and vaccination coverage percentage. Even though vaccination started almost at the same time in both countries, in Norway it was too late to be effective since the relative timing of the starting time of the vaccination, its location in the epidemic wave, is significant. For an efficient vaccination campaign the ratio *QV* should be small and even negative and in many countries the *QV* ratio was too high to be effective.

We presented simulations for vaccination coverage and the timing of the vaccination with respect to the peak of the epidemic to study their role in vaccination efficiency. We realized that on-time vaccinations considerably reduce the final value of *R*_*f*_, but these effects are practically too little to be observed on the shape of the normalized curve *R*(*t*)/*R*_*f*_. To study the effect of percentage of vaccination coverage, we compared no vaccination policy and 3% vaccination for the SIR model and realized that *R*_*f*_ is lower in 3% strategy than no vaccination policy even though final value of *S*(*t*) is more or less the same. Hungary, Ireland, Netherlands, Norway, and Sweden have vaccination coverage percentages above 20%, so in these countries vaccinations were considered to be effective on *R*_*f*_. To study the effect of the timing of pulse vaccination, we presented SIR model results of 30% vaccination coverage percentage starting early and late, one week earlier or later than the time *t*_*m*_, where *I*(*t*) for the no vaccination model reaches its maximum value. Based on these results, we see that vaccinations that start late have little effect on reductions of *R*_*f*_, and also even 2–4-week campaigns may be sufficient and campaigns that continue beyond the stabilization of *R*(*t*) are not effective for influenza type epidemics. To study the effect of vaccination coverage percentages on actual and normalized curves, we presented *R*(*t*) and *R*(*t*)/*R*_*f*_ curves for different vaccination coverage percentages and realized that percentage of vaccination had little effect on the shape of *R*(*t*). Low rates were practically unobservable in *R*(*t*)/*R*_*f*_ curves but at high vaccination percentage rates the effect on *R*(*t*)/*R*_*f*_ was a shift and a reduction in the curvature of the first turn for early vaccination timing and a reduction of the curvature of the second turn for late vaccination timing. Finally, SIR model simulations were used to show the relative improvements in *R*_*f*_ when different pulse vaccination strategies are used.

## 6. Conclusions

We have seen that healthcare practices and HI of countries as well as vaccination campaigns explain the variations among relative fatalities. On-time vaccinations have a considerable effect on reducing the ratio of individuals that are Removed after going through an infections cycle, *R*_*f*_; however, this effect is not practically observable in normalized time evolution curves *R*(*t*)/*R*_*f*_, especially at low vaccination rates. An efficient vaccination campaign should start early in the phases of the epidemic but does not need to continue over the peak of the epidemic. We recall that *R*_0_ can be estimated at the beginning of an epidemic; hence the peak of *I*(*t*) can be estimated without the vaccine intervention. Based on this pieces of information, the timing and the coverage percentage of the vaccination can be planned effectively.

As a tool for controlling the epidemic, the timing of the pulse vaccination is crucial. The simulations show the importance of the timing of the vaccination and show that vaccinations that start late have little effect in reducing *R*_*f*_. In order to be effective, vaccination should start in the early phases of the epidemic but does not need to continue over the peak of the epidemic. The comparison of the vaccination timings for Norway and Sweden is a good example for this situation. The simulation results presented in Section 4.4 support the importance of the timing in vaccination campaigns.

Our study is limited to what can be inferred from publicly available data; we used WISO reports of ECDC and restricted our investigation to European countries. These countries display relatively small variations in their demographic structures and healthcare systems; hence our conclusions should not be generalized worldwide.

## Figures and Tables

**Table 1:** Cumulative weekly fatalities due to A(H1N1) pandemic in Europe.

Week	1st day	Czech R.	Estonia	France	Germany	Greece	Hungary	Ireland	Lithuania	Netherlands	Norway	Romania	Slovenia	Sweden
36 (36/09)	30.08.2009	0	0	0	0	1	1	2	0	1	0	0	0	2
37 (37/09)	07.09.2009	0	0	0	0	1	1	2	0	4	2	0	0	2
38 (38/09)	14.09.2009	0	0	0	0	1	1	2	0	5	2	0	0	2
39 (39/09)	21.09.2009	0	0	0	0	1	1	2	0	4	2	0	0	2
40 (40/09)	28.09.2009	0	0	0	0	1	1	4	0	4	2	0	0	2
41 (41/09)	05.10.2009	0	0	0	0	1	1	4	0	4	2	0	0	2
42 (42/09)	12.10.2009	0	0	0	0	1	5	5	0	4	4	0	0	2
43 (43/09)	19.10.2009	1	0	0	0	1	3	9	0	6	7	0	0	2
44 (44/09)	26.10.2009	1.6	0	10.3	4.3	1	3.3	11.6	0	23.6	10.6	0	0	4.6
45 (45/09)	02.11.2009	2.3	0	20.6	8.6	1	3.6	14.3	0	41.3	14.3	0	0	7.3
46 (46/09)	09.11.2009	3	0	31	13	1	4	17	0	59	18	0	0	10
47 (47/09)	16.11.2009	6	1	46	13	1	8	17	1	32	24	1	2	15
48 (48/09)	23.11.2009	12	2	92	66	16	13	18	2	37	25	3	5	15
49 (49/09)	30.11.2009	27	3	92	94	33	23	19	6	47	27	12	10	17
50 (50/09)	07.12.2009	27	5	150	119	49	36	22	10	54	29	18	10	19
51 (51/09)	14.12.2009	46	6	150	123	49	45	22	14	52	29	32	10	20
52 (52/09)	21.12.2009	59.5	8.5	182	140	61	51	22	15	53	29	47.5	12.5	21
53 (53/09)	28.12.2009	73	11	214	157	73	57	22	16	54	29	63	15	22
54 (01/10)	04.01.2010	83	11	243	176	89	70	22	18	54	29	84	16	22
55 (02/10)	11.01.2010	83	13	261	187	98	94	22	19	56	29	104	16	22
56 (03/10)	18.01.2010	91	14	275	199	106	107	22	21	56	29	110	19	23
57 (04/10)	25.01.2010	91	14	285	215	118	112	22	22	56	29	116	19	24
58 (05/10)	01.02.2010	94	15	289	225	123	119	22	22	57	29	120	19	24
59 (06/10)	08.02.2010	96	15	296	235	130	121	22	22	57	29	121	19	24
60 (07/10)	15.02.2010	97	16	302	239	135	124	22	23	58	29	122	19	24
61 (08/10)	22.02.2010	97	18	306	243	137	129	22	23	58	29	122	19	24
62 (09/10)	01.03.2010	97	19	308	243	138	130	23	23	58	29	122	19	24
63 (10/10)	08.03.2010	98	19	309	243	139	130	24	23	59	29	122	19	24
64 (11/10)	13.03.2010	98	19	310	250	139	130	24	23	60	29	122	19	24
65 (12/10)	22.03.2010	98	19	311	252	140	130	25	23	61	29	122	19	24
66 (13/10)	29.03.2010	98	19	312	254	140	132	24	23	62	29	122	19	24
67 (14/10)	05.04.2010	98	19	312	253	141	133	25	23	61	29	122	19	24
68 (15/10)	12.04.2010	98	19	312	253	141	134	26	23	61	29	122	19	24

**Table 2:** Demographic information.

Country	*D*_*f*_	*N*	*A*	*d* = *N*/*A*	*N* < 65 (%)	(*D*_*f*_/*N*)10^3^	HDI	HI	*λ*
Czech Rep.	98	10 467	78 866	132.7	85.1	9.4	0.841	0.901	49.45
Estonia	19	1 340	45 226	29.63	82.9	14	0.812	0.851	59.00
France	312	64 367	643 548	100.0	83.3	4.9	0.872	0.976	46.00
Germany	253	82 002	357 021	229.7	79.6	3.1	0.885	0.953	51.00
Greece	141	11 260	131 940	85.34	81.3	13	0.855	0.945	39.00
Hungary	134	10 031	93 030	107.8	83.6	13	0.805	0.853	47.00
Ireland	26	4 450	70 280	63.32	88.9	5.8	0.895	0.955	53.00
Lithuania	23	3 349	65 200	51.37	84.0	6.9	0.783	0.824	56.00
Netherlands	61	16 485	41 526	397.0	85.0	3.7	0.890	0.955	52.30
Norway	29	4 799	385 252	12.46	85.3	6.0	0.938	0.966	62.00
Romania	122	21 498	238 391	90.18	85.1	5.7	0.767	0.842	46.00
Slovenia	19	2 032	20 253	100.3	83.6	9.4	0.828	0.931	46.00
Sweden	24	9 256	449 964	20.57	82.2	2.6	0.885	0.970	62.00

Note: *D*_*f*_: total fatality; *N*: population in thousands; *A*: area (km^2^); *d*: population density (thousand/km^2^); *N* < 65 (%): percentage of 0–64 years; (*D*_*f*_/*N*)10^3^: relative fatalities; HDI: Human Development Index; HI: health index; *λ*: average latitude degree north.

**Table 3:** Vaccination coverage.

Country	*t*_*i*_	*t*_1_	*t*_*e*_	*t*_2_	Δ*T*	Δ*V*	*QV*	*V*_*f*_
Czech Rep.	41	48	64	76	23	7	0.30	0.6
Estonia	45	51	63	86	18	6	0.33	3
France	42	43	67	86	25	1	0.04	8
Germany	42	44	67	85	25	2	0.08	8
Greece	46	47	68	86	22	1	0.04	3
Hungary	40	40	63	86	23	0	0.00	27
Ireland	38	43	51	86	13	5	0.38	23
Lithuania	45	53	61	86	16	8	0.50	—
Netherlands	41	44	61	56	20	3	0.15	30
Norway	40	43	51	66	11	3	0.27	45
Romania	45	48	61	77	16	3	0.19	9
Slovenia	45	44	57	58	12	−1	−0.08	5
Sweden	42	42	58	86	16	0	0.00	59

Note: *t*_*i*_: the onset of the epidemic wave estimated as the week before the first fatality; *t*_1_: the first week of vaccination; *t*_*e*_: the end of the epidemic wave estimated as the week after the last fatality; *t*_2_: the last week of vaccination (counted from the beginning of 2009); Δ*T*: the duration of the epidemic wave, Δ*T* = *t*_*e*_ − *t*_*i*_; Δ*V*: the time span between the onset of the epidemic pulse and the starting of the pulse vaccination, Δ*V* = *t*_1_ − *t*_*i*_; *QV* = Δ*V*/Δ*T*: the relative timing of the vaccination campaign within the epidemic pulse (a negative or small positive value indicated on time vaccination campaign); *V*_*f*_: total vaccination percentage.
